# Host Factors and Biomarkers Associated with Poor Outcomes in Adults with Invasive Pneumococcal Disease

**DOI:** 10.1371/journal.pone.0147877

**Published:** 2016-01-27

**Authors:** Shigeo Hanada, Satoshi Iwata, Kazuma Kishi, Miyuki Morozumi, Naoko Chiba, Takeaki Wajima, Misako Takata, Kimiko Ubukata, The Invasive Pneumococcal Diseases Surveillance Study Group

**Affiliations:** 1 Department of Respiratory Medicine, Respiratory Center, Toranomon Hospital, Tokyo, Japan; 2 Department of Infectious Diseases, Keio University School of Medicine, Tokyo, Japan; 3 Okinaka Memorial Institute for Medical Research, Tokyo, Japan; 4 Department of Microbiology, School of Pharmacy, Tokyo University of Pharmacy and Life Sciences, Tokyo, Japan; University of Leicester, UNITED KINGDOM

## Abstract

**Background:**

Invasive pneumococcal disease (IPD) causes considerable morbidity and mortality. We aimed to identify host factors and biomarkers associated with poor outcomes in adult patients with IPD in Japan, which has a rapidly-aging population.

**Methods:**

In a large-scale surveillance study of 506 Japanese adults with IPD, we investigated the role of host factors, disease severity, biomarkers based on clinical laboratory data, treatment regimens, and bacterial factors on 28-day mortality.

**Results:**

Overall mortality was 24.1%, and the mortality rate increased from 10.0% in patients aged ˂50 years to 33.1% in patients aged ≥80 years. Disease severity also increased 28-day mortality, from 12.5% among patients with bacteraemia without sepsis to 35.0% in patients with severe sepsis and 56.9% with septic shock. The death rate within 48 hours after admission was high at 54.9%. Risk factors for mortality identified by multivariate analysis were as follows: white blood cell (WBC) count <4000 cells/μL (odds ratio [OR], 6.9; 95% confidence interval [CI], 3.7–12.8, *p* < .001); age ≥80 years (OR, 6.5; 95% CI, 2.0–21.6, *p* = .002); serum creatinine ≥2.0 mg/dL (OR, 4.5; 95% CI, 2.5–8.1, *p* < .001); underlying liver disease (OR, 3.5; 95% CI, 1.6–7.8, *p* = .002); mechanical ventilation (OR, 3.0; 95% CI, 1.7–5.6, *p* < .001); and lactate dehydrogenase ≥300 IU/L (OR, 2.4; 95% CI, 1.4–4.0, *p* = .001). Pneumococcal serotype and drug resistance were not associated with poor outcomes.

**Conclusions:**

Host factors, disease severity, and biomarkers, especially WBC counts and serum creatinine, were more important determinants of mortality than bacterial factors.

## Introduction

Invasive pneumococcal disease (IPD) contributes considerably to morbidity and mortality worldwide despite availability of effective vaccines and antimicrobial agents. Incidence of IPD is greatest in adults ≥65 years old, in children <2 years old [1], and in persons with certain chronic diseases or conditions [2–7]. Overall mortality rates for patients with IPD have consistently ranged from 9.3% to 17%, with two-thirds of deaths occurring within the first 3 days following admission to a hospital [8–11].

IPD has become a serious problem in Japan as well as other developed countries, largely because of increasingly aging populations. In Japan, the percentage of the population ≥65 years reached 26.0% in 2014 (http://www.stat.go.jp/english/index.htm). However, little information regarding the incidence of IPD in Japan has been reported. Furthermore, Japan only began promoting routine vaccination of adults who are ≥65 years old in 2014, and the Japanese vaccination rate before 2014 was considerably less than 20%.

Multiple studies have reported that risk factors significantly associated with death in patients with IPD include age [9, 10, 12, 13], disease severity [8, 9, 12, 13], presence of underlying diseases or immunosuppression [8–10], and strains with reduced susceptibility to β-lactam agents [14]. While the extent to which host and bacterial factors each contribute to risk of mortality is difficult to assess, this information would greatly enhance understanding of the pathophysiology of this severe disease and enhance efforts to improve outcomes.

In the present study, we conducted a large-scale surveillance study of IPD in Japanese adults to evaluate the relative contributions of host factors, laboratory findings, disease severity, treatment regimens, and bacterial factors to 28-day mortality after admission.

## Materials and Methods

### Ethics statement

The study complied with the tenets of the Declaration of Helsinki and the Guidelines for Epidemiologic Studies from the Japanese Ministry of Health, Labour, and Welfare. The study protocol was approved by the institutional review board of Kitasato Institute of Life Sciences, Kitasato University, and the requirement for written informed consent from the patients was waived due to the use of anonymized stored samples and data. In addition, the laboratory or hospital director for each center provided written permission to participate in this surveillance.

### Study design

This prospective observational multicenter cohort study was performed at the Laboratory of Molecular Epidemiology for Infectious Agents, Kitasato Institute for Life Sciences, Kitasato University. Among 1317 patients with IPD who were admitted to 341 hospitals throughout Japan between April 2010 and March 2013, adults at least 18 years old numbered 715. Patients with meningitis (n = 127) were excluded from this study due to their significant long-term morbidity; approximately 28.3% of these patients develop severe chronic neurologic sequelae such as altered consciousness, seizures, focal neurologic deficits, hearing loss, and intellectual impairment. Therefore, we analyzed these patients separately in another study that was based on the other clinically-relevant outcomes, such as favorable neurologic function. Furthermore, we excluded patients with focal infections, such as arthritis, cellulitis, and spondylitis, which were outside the scope of the present survey. Moreover, we excluded patients with insufficient clinical data. After these exclusions, the remaining 506 patients were followed for 28 days after their admission (S1 Fig). More details of study methods have been described previously [15, 16].

### Definitions

IPD was defined as an infection confirmed by isolation of *S*. *pneumoniae* from normally sterile clinical samples such as blood and pleural fluid. Underlying chronic disease was defined as presence of cardiovascular disease, liver disease (e.g., chronic hepatitis or cirrhosis that was secondary to alcohol abuse or viral infection), renal disease, lung disease, cancer, or diabetes mellitus. Immunosuppressed patients were defined as those who had undergone splenectomy, had a hematologic or autoimmune disorder, received a transplant, or underwent cancer chemotherapy within 4 weeks before the onset of IPD. Disease severity was assessed using diagnostic criteria for sepsis [17] at the time of admission. Antibiotic treatment regimens were placed into 5 categories: penicillins, first or second-generation cephalosporins, third or fourth-generation cephalosporins, carbapenems, and others.

### Serotypes and antimicrobial drug-resistant genotypes

Serotypes of all isolates were determined by the capsular quellung reaction using antiserum purchased from the Statens Serum Institute (Copenhagen, Denmark). We classified these pneumococci into 3 serotype categories: 7-valent pneumococcal conjugate vaccine (PCV7) serotypes; 13-valent pneumococcal conjugate vaccine (PCV13) plus 23-valent pneumococcal polysaccharide vaccine (PPSV23) serotypes (except for PCV7 serotypes); and non-vaccine serotypes. Alterations in 3 penicillin-binding protein (PBP) genes mediating β-lactam resistance in *S*. *pneumoniae*, *pbp1a*, *pbp2x*, and *pbp2b* genes encoding enzymes of PBP1A, PBP2X, and PBP2B, respectively, were identified by real-time PCR methods [15, 16]. Genotype (g) based on molecular analysis is represented as penicillin-susceptible *S*. *pneumoniae* (gPSSP) possessing 3 normal *pbp* genes; penicillin-intermediate *S*. *pneumoniae* (gPISP), further classified as gPISP (*pbp2x*), gPISP (*pbp1a+pbp2x*), or gPISP (*pbp2x+pbp2b*); or penicillin-resistant *S*. *pneumoniae* (gPRSP), possessing all 3 abnormal *pbp* genes.

### Statistical analysis

Fatalities occurring within 28 days after admission were defined as deaths caused by or related to pneumococcal infection. Categorical variables were tested using the χ^2^ test. Continuous variables were compared using the *t* test. Cut-offs for laboratory values were determined by the distribution of each biomarker. The Kaplan-Meier method was used to determine associations between disease severity and survival. Logistic regression modelling was performed for variables selected for inclusion in multivariate models; for stepwise, backward elimination, and forward selection methods, factors associated with mortality with *p* values < .30, < .30, and < .35 for were retained in the models, respectively. Careful consideration was given to relationships between different variables using Pearson correlation analysis, which determined variables to be included in the multivariate models to perform proper adjustment for confounders. Highly correlated variables (≥0.5) were excluded from multivariate analyses. Statistical significance was defined as *p* < .05. All analyses were performed using SAS software (version 9.3, SAS Institute, Cary, NC).

## Results

### Patient characteristics

Demographic and clinical features of patients and characteristics of causative agent are summarized in Table 1. The overall 28-day mortality rate among IPD patients was 24.1% (n = 122). The mean patient age was 68.7 years (median, 71 years; range, 21–97 years) and 74.3 years (median, 76 years; range, 24–97 years) in surviving and deceased patients, respectively.

**Table 1:** Characteristics of adults with invasive pneumococcal disease associated with fatal outcome.

Characteristic	Overall (n = 506)	Survived (n = 384)	Died (n = 122)	Univariate analysis	*p* value ^j^
	n (%)	n	n	OR (95% CI) ^k^	
**Age**					
<50 y	50 (9.9)	45	5	1	-
50–64 y	105(20.8)	87	18	1.9 (0.6–5.3)	NS
65–79 y	206 (40.7)	155	51	3.0 (1.1–7.9)	.029
≥80 y	145 (28.7)	97	48	4.5 (1.7–11.9)	.003
**Gender** ^a^					
Female	177 (35.0)	135	42	1	-
Male	315 (62.3)	236	79	1.1 (0.7–1.7)	NS
**Underlying diseases and conditions**					
With any underlying diseases	458 (90.5)	344	114	1.7 (0.8–3.6)	NS
Malignancy	119 (23.5)	91	28	1.0 (0.6–1.6)	NS
Diabetes mellitus	92 (18.2)	68	24	1.1 (0.7–1.9)	NS
Cardiovascular disease	92 (18.2)	66	26	1.3 (0.8–2.2)	NS
Immunosuppression	64 (12.7)	53	11	0.6 (0.3–1.2)	NS
Liver disease^b^	50 (9.9)	33	17	1.7 (0.9–3.2)	.088
Lung disease^c^	38 (7.5)	34	4	0.3 (0.1–1.0)	NS
Renal disease	38 (7.5)	30	8	0.8 (0.4–1.9)	NS
**Clinical manifestations**					
Pneumonia ^d^	356 (70.4)	273	83	1	-
Bacteraemia without focus	150 (29.6)	111	39	1.2 (0.7–1.8)	NS
**Disease Severity** ^e^					
Bacteraemia without sepsis	104 (20.5)	91	13	1	-
Sepsis	167 (33.0)	153	14	0.6 (0.3–1.4)	NS
Severe sepsis	177 (35.0)	115	62	3.8 (2.0–7.3)	< .001
Septic shock	58 (11.5)	25	33	9.2 (4.2–20.1)	< .001
**Interventions to restore perfusion** ^f^	49 (9.7)	27	22	2.9 (1.6–5.3)	< .001
**Mechanical ventilation**	92 (18.2)	52	40	3.1 (1.9–5.0)	< .001
**Genotypic penicillin resistance** ^g^					
gPSSP	83 (16.4)	67	16	1	-
gPISP	296 (58.5)	223	73	1.4 (0.7–2.5)	NS
gPRSP	127 (25.1)	94	33	1.5 (0.7–2.9)	NS
**Serotype** (n = 503)					
PCV7 serotype	183 (36.4)	139	44	1	-
PCV13 and PPSV23 ^h^	232 (46.1)	182	50	0.9 (0.5–1.4)	NS
Non-vaccine serotypes	88 (17.5)	60	28	1.5 (0.8–2.6)	NS
**Initial antibiotic therapy** ^i^ (n = 482)					
Penicillin	23 (4.6)	18	5	1	-
1st or 2nd cephalosporins	64 (13.3)	51	13	1.1 (0.5–2.2)	NS
3rd or 4th cephalosporins	99 (20.5)	87	12	0.6 (0.3–1.2)	NS
Carbapenems	167 (34.6)	135	32	2.3 (1.4–4.0)	.002
Others	129 (26.8)	83	46	1.2 (0.4–3.4)	NS

^a^ Excluded unknown.

^b^ Includes chronic hepatitis or cirrhosis that was secondary to alcohol abuse or viral infection

^c^ Includes chronic obstructive pulmonary disease, bronchial asthma, interstitial lung disease, and bronchiectasis.

^d^ Includes empyema (n = 16) and pleuritis (n = 14).

^e^ Classified according to international guidelines for management of severe sepsis and septic shock [16].

^f^ Includes modalities such as vasopressor therapy, inotropic therapy, and/or hemodynamic support, depending on response to fluid resuscitation.

^g^ gPSSP, genotypic penicillin (PC)-susceptible *S*. *pneumoniae*; gPISP, genotypic PC- intermediate resistant *S*. *pneumoniae*; gPRSP, genotypic PC-resistant *S*. *pneumoniae*.

^h^ PCV13 plus PPSV23 serotypes except for PCV7 serotype 23-valent pneumococcal polysaccharide vaccine.

^i^ 1st or 2nd cephalosporins, first- or second-generation cephalosporins; 3rd or 4th cephalosporins, third- or fourth-generation cephalosporins.

^j^ NS, not significant (*p*≥.05).

^k^ OR, odds ratio; CI, confidence interval.

The mortality rate increased significantly with age, from 10.0% (n = 5) in patients <50 years to 17.1% (n = 18) in those aged 50–64 years, 24.8% (n = 51) in those aged 65 to 79 years, and 33.1% (n = 48) in those ≥80 years. The majority of the patients had some underlying disease, but only liver disease was moderately associated with mortality. The most common clinical manifestations of IPD were pneumonia and bacteraemia with no identified focus of origin. Mechanical ventilation and interventions to restore perfusion were required significantly more often in patients who died than in those who survived. Frequencies of strains with reduced susceptibility to penicillin were similar in these 2 groups. No significant difference in mortality was evident between infections caused by vaccine and by non-vaccine serotypes. The proportion of patients receiving antibiotics as initial therapy differed slightly between the 2 groups, since empiric antibiotic treatment was influenced by apparent disease severity. However, this difference was not statistically significant on the multivariate analysis.

### Mortality rate by age group and disease severity

The mortality rate increased with severity of sepsis, from 12.5% (n = 13) and 8.4% (n = 14) among patients with bacteraemia without or with sepsis, respectively, to 35.0% (n = 62) and 56.9% (n = 33) among patients with severe sepsis or septic shock, respectively (Table 1).

**Fig 1:**
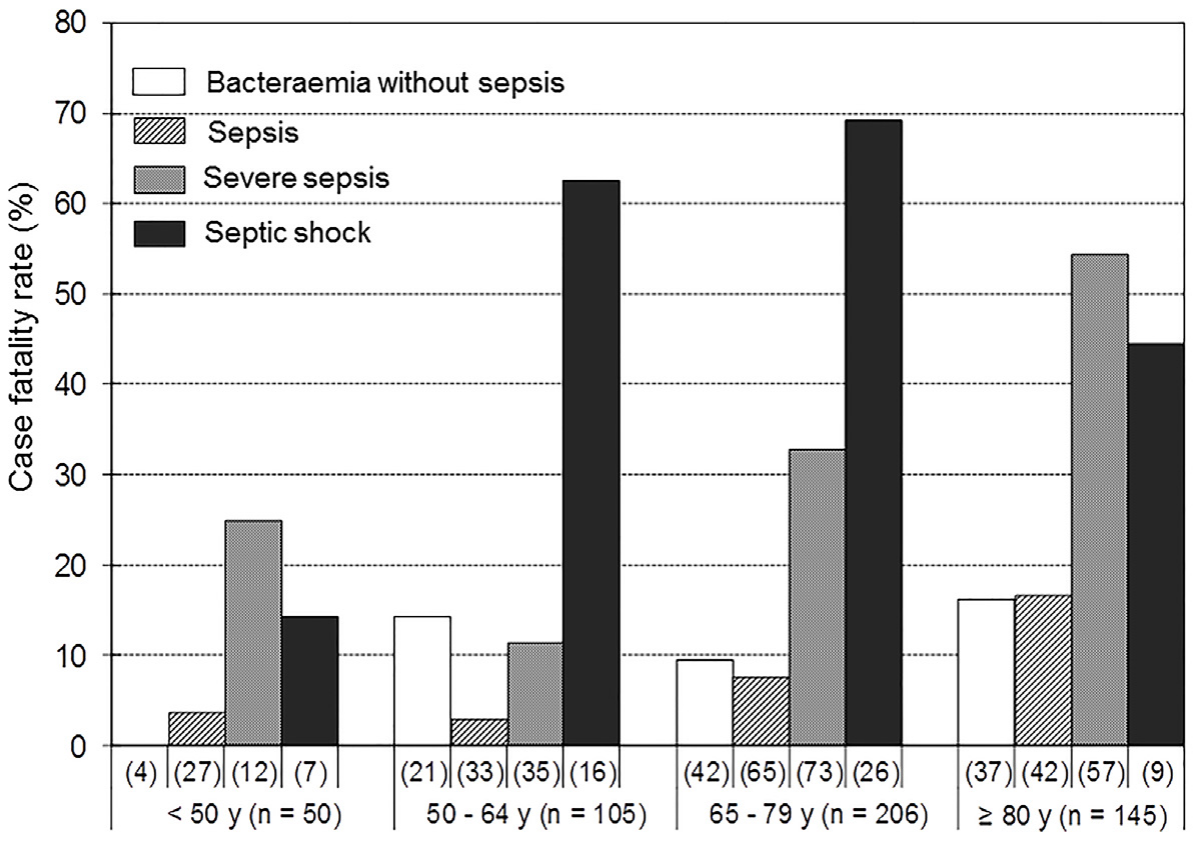
Case fatality rates by age groups and disease severity.

Within each age group (<50 years, 50–64 years, 65–79 years, and ≥80 years), the mortality rate increased with the severity of sepsis. As expected, the mortality rate within each sepsis severity category was significantly higher in older age groups (Fig 1).

### Association of laboratory findings with outcome

Laboratory findings associated with fatal outcome in adults with IPD are summarized in Table 2. On admission, median white blood cell (WBC) and platelet counts were lower in patients who died than in those who survived, while concentrations of aspartate aminotransferase (AST), alanine aminotransferase (ALT), blood urea nitrogen (BUN), serum creatinine (Cr), creatine kinase (CK), and lactate dehydrogenase (LDH) were higher in patients who died than in those who survived. Leukopenia (WBC <4000 cells/μL), thrombocytopenia (PLT <10.0×10^4^/μL), and organ injury (as evidenced by AST ≥100 IU/L, ALT ≥100 IU/L, BUN ≥40mg/dL, Cr ≥2.0mg/dL, CK ≥200 IU/L, and LDH ≥300 IU/L) were more frequent among patients who died than in survivors.

**Table 2:** Laboratory findings associated with fatal outcome in adults with invasive pneumococcal disease.

Characteristic ^a^	Survived (n = 384)		Died (n = 122)		Univariate analysis	*p* value
	No. of assessed	Median (range)n (%)	No. of assessed	Median (range) n (%)	OR (95%CI)	
WBC (×10^3^ cells/μL)	384	12.5 (0.1–55.8)	122	6.8 (0.2–47.7)		
(<4.0×10^3^ cells/μL)		39 (10.2)		41 (33.6)	4.5 (2.7–7.4)	< .001
PLT (×10^4^ cells/μL)	383	18.2 (0.8–69.9)	122	14.3 (0.7–59.3)		
(<10.0×10^4^ cells/μL)		56 (14.6)		34 (27.9)	2.3 (1.4–3.7)	.001
CRP (mg/dL)	379	20.5 (0.5–60.1)	121	23.1 (0.2–89.6)		
(≥20 mg/dL)		194 (51.2)		73 (60.3)	1.5 (1.0–2.2)	.079
AST (IU/L)	382	31 (9–2,757)	121	63 (8–7,953)		
(≥100 IU/L)		46 (12.0)		41 (33.9)	3.7 (2.3–6.1)	< .001
ALT (IU/L)	380	22 (2–961)	121	28 (8–2,510)		
(≥100 IU/L)		22 (5.8)		24 (19.8)	4.0 (2.2–7.5)	< .001
BUN (mg/dL)	379	24 (4–154)	122	48.5 (6–151)		
(≥40 mg/dL)		89 (23.4)		76 (62.3)	5.4 (3.5–8.4)	< .001
Cr (mg/dL)	381	0.9 (0.2–12.9)	121	1.7 (0.5–7.7)		
(≥2.0 mg/dL)		46 (12.1)		44 (36.4)	4.2 (2.6–6.7)	< .001
CK (IU/L)	309	86 (2–21,890)	111	182 (14–77,480)		
(≥200 IU/L)		79 (25.6)		54 (48.6)	2.8 (1.8–4.3)	< .001
LDH (IU/L)	367	246 (4–4,604)	115	314 (26–16,675)		
(≥300 IU/L)		118 (32.1)		61 (53.0)	2.4 (1.6–3.7)	< .001

^a^ Abbreviations of characteristics;WBC, white blood cell count; PLT, platelets; CRP, C-reactive protein; AST, aspartate aminotransferase; ALT, alanine aminotransferase; BUN, blood urea nitrogen; Cr, creatinine; CK, creatine kinase; LDH, lactate dehydrogenase; OR, odds ratio; CI, confidence interval.

### Deaths in relation to the time elapsed after admission

**Fig 2:**
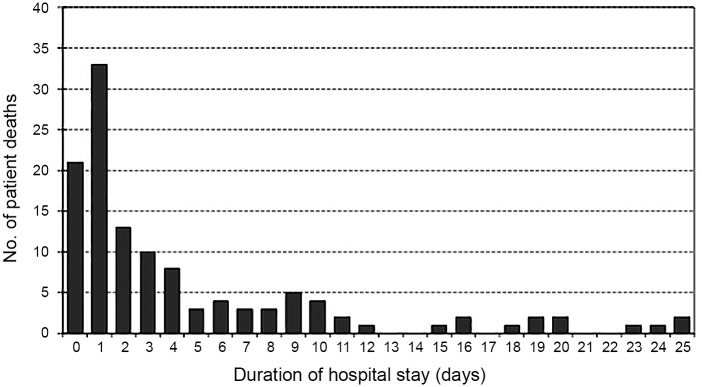
Distribution of deaths in patients with invasive pneumococcal disease in relation to the time elapsed after admission to the hospital.

**Fig 3:**
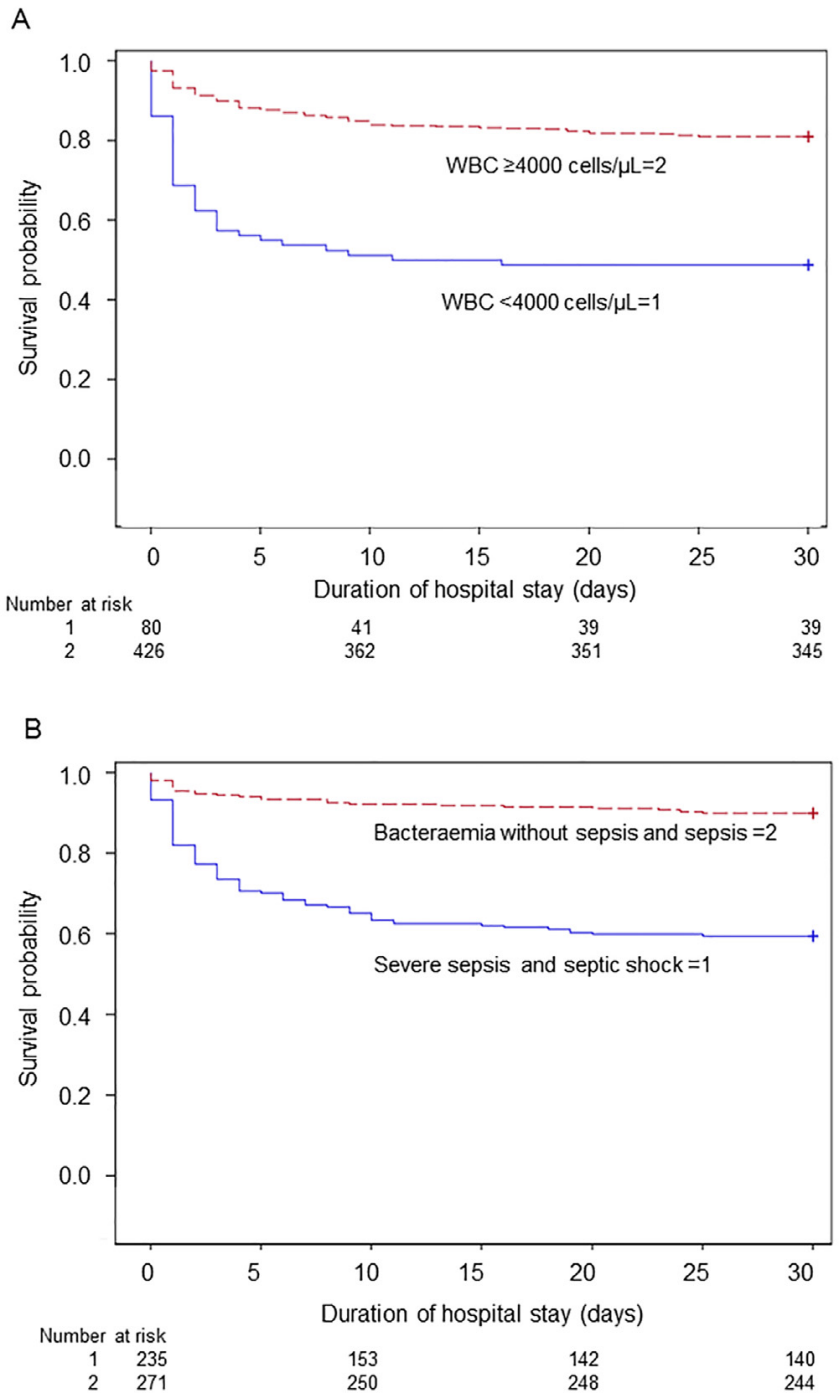
Kaplan-Meier estimate of the probability of 28-day survival. **(A)** Patients with leukopenia on admission had a higher risk for death, compared to patients without leukopenia. The majority of deaths occurred within the first 5 days after admission to the hospital (*p* < .001 by the log-rank test). WBC, white blood cell count. **(B)** Patients with severe sepsis or septic shock compared to patients with bacteraemia without sepsis or sepsis on admission. Mortality increased with severity of sepsis (*p* < .001 by the log-rank test).

As shown in Fig 2, 54.9% (n = 67) of patients died within 48 hours and 72.1% (n = 88) died within 5 days. The likelihood of 28-day survival was lower among IPD patients with leukopenia (WBC <4000 cells/μL) on admission than among patients without leukopenia (*p* < .001 by the log-rank test; Fig 3A). Critical illness (i.e., severe sepsis and septic shock) was also significantly associated with mortality (*p* < .001 by the log-rank test; Fig 3B). Kaplan-Meier analysis indicated that the risk of death was highest during the first 5 days after admission.

### Prognostic factors related to 28-day mortality

Pearson correlation analysis was performed to determine whether a correlation existed between the serum concentrations of biomarkers in patients with IPD (S1 and S2 Tables). Significant correlations were noted between AST, ALT, CK, and LDH, as well as between BUN and Cr. Based on these analyses, clinical relevance, and odds ratios (OR) on univariate analysis for each variable, we selected LDH and Cr for multivariate analysis.

Multivariate analysis shown in Table 3 revealed that age ≥80 years, presence of underlying liver disease, mechanical ventilation, WBC <4000 cells/μL, Cr ≥2.0 mg/dL, and LDH ≥300 IU/L were statistically significant risk factors for IPD mortality. Bacterial serotypes, genotypic penicillin resistances, and types of initial antibiotic therapies did not appear to influence risk of mortality.

**Table 3:** Risk factors associated with mortality within 28 days among patients with invasive pneumococcal disease by multivariate logistic regression analysis.

Characteristic	Survived (n = 384)	Died (n = 122)	Multivariate analysis ^e^	*p* value ^f^
	n (%)	n (%)	OR (95% CI)	
**Age groups**				
<50 y	45 (11.7)	5 (4.1)	1	-
50–64 y	87 (22.7)	18 (14.8)	1.6 (0.5–5.6)	NS
65–79 y	155 (40.4)	51 (41.8)	3.0 (0.9–9.6)	NS
≥80 y	97 (25.3)	48 (39.3)	6.5 (2.0–21.6)	.002
**Underlying disease**				
Liver disease^a^	33 (8.6)	17 (13.9)	3.5 (1.6–7.8)	.002
Lung disease^b^	34 (8.9)	4 (3.3)	0.4 (0.1–1.4)	NS
**Mechanical ventilation**	52 (13.5)	40 (32.8)	3.0 (1.7–5.6)	< .001
**Laboratory findings** ^c^				
WBC (<4.0×10^3^ cells/μL)	39 (10.2)	41 (33.6)	6.9 (3.7–12.8)	< .001
Cr (≥2.0 mg/dL)	46 (12.1)	44 (36.4)	4.5 (2.5–8.1)	< .001
LDH (≥300 IU/L)	118 (32.1)	61 (53.0)	2.4 (1.4–4.0)	.001
**Serotype** ^d^				
PCV7 serotypes	139 (36.2)	44 (36.1)	1	-
PCV13 and PPSV23 serotypes	182 (47.4)	50 (41.0)	0.8 (0.5–1.5)	NS
Non-vaccine serotypes	60 (15.6)	20 (23.0)	(0.8–3.0)	NS

^a^ Includes chronic hepatitis or cirrhosis that was secondary to alcohol abuse or viral infection.

^b^ Includes chronic obstructive pulmonary disease, bronchial asthma, interstitial lung disease, and bronchiectasis.

^c^ Laboratory findings; WBC, white blood cell count; Cr, creatinine; LDH, lactate dehydrogenase.

^d^ Serotype; PCV7, 7-valent pneumococcal conjugate vaccine; PCV13, 13-valent pneumococcal conjugate vaccine; PPSV23, 23-valent pneumococcal polysaccharide vaccine.

^e^ Multivariate analysis; OR, odds ratio; CI, confidence interval.

^f^ NS, not significant (*p* ≥ .05).

Receiver operating characteristic analyses of the sensitivity and specificity of 28-day mortality prediction showed an area under the curve of 0.819 for these significant prognostic factors (S2 Fig).

## Discussion

In the most extensive surveillance of adults with IPD in Japan, we revealed an overall 28-day mortality rate of 24.1%. Mortality was significantly associated with host risk factors, such as age (≥80 years) and presence of underlying liver disease, but showed no association with bacterial factors such as serotype or antibiotic resistance; these data are consistent with those of previous studies [9, 10, 12, 13]. Despite good access to medical care in Japan, the mortality rate in the present study was higher than those reported by previous investigators [8–11]. One explanation could be that this study included relatively older patients than in previous studies. Aging-related changes in immune function, referred to as immunosenescence, cause a decrease in adaptive immune function [18]. A recent study of cell responses to pneumococci *in vivo* reported age-related impairment of alveolar macrophages and Toll-like receptor levels [19]. Furthermore, aging is associated with a procoagulant state and mitochondrial damage that cause cellular apoptosis during sepsis [20]. We also found that chronic liver disease was associated with an increased risk of mortality, which confirms similar findings among patients with pneumococcal infections and chronic liver diseases, in the absence of other causes of immunodeficiency [7, 21, 22]. Moreover, bacterial infection and sepsis have been recognized as a distinct stage in the natural progression of chronic liver disease, as they accelerate organ failure and ultimately lead to death. A previous study has demonstrated that abnormal immunity may be attributable to liver disease, which affects innate and adaptive immunity, and this abnormal immunity might increase the risk of infection, which might subsequently increase the risk of systemic complications [21].

In the present study, we found that the bacterial serotype groups and genotypic penicillin resistance did not significantly affect mortality, as described previously [9, 10]. Nevertheless, one meta-analysis has reported significant differences in the risk ratios for death among patients infected with pneumococcal serotypes [23]. Therefore, debate continues regarding the extent to which pneumococcal serotypes, penicillin resistance, and details of early antibiotic administration affect outcome of IPD.

Disease severity on admission was an independent risk factor significantly associated with mortality in our study. Similarly, a hospital-based cohort study found the 30-day mortality rate to increase with sepsis severity, from 4% and 6% among patients with no sepsis or sepsis, respectively, to 18% among patients with severe sepsis or septic shock (*p* = .005) [13]. Other studies reported high mortality rates among critically ill patients, regardless of the choice of antibiotics [8, 12]. Therefore, it appears that the critical illness can often overwhelm any salutary effects of antibiotic activity predicted by in vitro testing.

In this study, WBC <4000 cells/μL, serum Cr concentrations ≥2.0 mg/dL, and LDH concentrations ≥300 IU/L were found to be important biomarkers showing strong correlation with poor outcome. Similarly, one previous study reported that patients hospitalized because of pneumonia had increased mortality rates in the presence of leukopenia [24]. In addition, sepsis is often accompanied by acute renal failure, which further increases the likelihood of death. Acute tubular necrosis resulting from sepsis-induced tissue hypoperfusion and/or hypoxemia may contribute to renal injury. Furthermore, international guidelines [17] for the management of sepsis particularly emphasize leukopenia (WBC <4000 cells/μL), acute oliguria, and creatinine elevation. These guidelines also define severe sepsis as sepsis plus sepsis-induced organ dysfunction or tissue hypoperfusion, which is exemplified by various laboratory findings, such as Cr exceeding 2.0 mg/dL. Moreover, we found that serum concentrations of LDH, which is released from cells in response to cytoplasmic membrane damage, were increased in patients with poor outcomes. Thus, these high concentrations of LDH may reflect systemic tissue injury caused by hypoperfusion, typically indicating damage to multiple organs rather than a specific diagnosis. Several studies have found increased serum LDH concentrations in patients with *Pneumocystis* pneumonia [25, 26] and *Mycoplasma pneumoniae* pneumonia [27], which is likely due to pathogen-induced lung injury.

To reduce the prevalence of pneumococcal infection and decrease the mortality rate of IPD, two types of pneumococcal vaccines (PPSV23 [28] and PCV13 [29]) are currently approved for use in adults according to a recent update from the Advisory Committee on Immunization Practices [30]. A recent clinical trial with PCV13 in adults aged ≥65 years reported an efficacy of 46% against vaccine-type pneumococcal pneumonia, 45% against vaccine-type nonbacteraemic pneumococcal pneumonia, and 75% against vaccine-type IPD [31]. However, further studies are needed to determine the optimal vaccine-specific indications, schedules, immunogenicity differences among vaccines, and indirect effects on adults after childhood vaccination in Japan.

Our study has several limitations. First, 46.5% of the patients with IPD had severe sepsis or septic shock at admission, and we were often unable to obtained detailed clinical information regarding disease severity, such as the first symptom and clinical progression. However, these factors may be related to the risk of death, and future studies should evaluate the characteristics at clinical presentation and the time from the first symptom to antibiotic therapy. Second, due to our incomplete disease severity data, it is possible that we underestimated the number of patients with sepsis or organ failure. However, the number of patients who were excluded from this study by insufficient data was relatively low (7.3%). Therefore, the result would be less modifiable by this exclusion. Third, we categorized the pneumococcal serotypes into three groups (PCV7 serotypes, PCV13 and PPSV23 serotypes, and non-vaccine serotypes), as our sample size was underpowered to compare the mortality risk of each individual serotype. Fourth, we did not evaluate the vaccination rate. However, in Japan, routine PCV13 vaccination has been officially promoted for <5-year-old children since November 2013, and PPSV23 vaccination has been recommended for routine administration to ≥65-year-old individuals since October 2014. Nevertheless, the PPSV23 vaccination rates among these individuals remained <20% during the course of the present study, and we found no evidence that vaccination influenced the serotype-specific mortality among the older patients. Therefore, it appears unlikely that the vaccination rates significantly influenced our findings.

Finally, resource limitations in some centers may have prevented physicians from performing standard practice, and we could not control for the types of antibiotics that were administered and the criteria for admission to the intensive care units. Therefore, although advanced medical care in Japan is provided through a government-administered universal health insurance system, access to better primary care may help further reduce the rates of IPD-related morbidity and mortality.

To improve outcomes, policymakers should consider new strategies to promote vaccination among the aging population. Moreover, clinicians should seek avenues to improve their management of these critically ill patients, and microbiologists should continue performing research that aims to further our understanding of the host-pathogen relationship in IPD.

## Supporting Information

S1 Fig Flow chart indicating assessment and follow-up of patients. Among 1317 patients with IPD who were admitted to 341 hospitals throughout Japan between April 2010 and March 2013, adults at least 18 years old numbered 715. Patients with meningitis (n = 127) and focal infections such as arthritis, cellulitis, and spondylitis, that were beyond the limits of this survey of IPD (n = 30) were not considered and those with insufficient clinical data (n = 52), were also excluded from this study. After such exclusions, 506 remaining patients followed for 28 days after admission.
(DOCX)

S2 Fig Receiver operating characteristic (ROC) curve for 28-day mortality. Predictors of age ≥80 years, underlying liver disease, mechanical ventilation, white blood cell count <4000 cells/μL, creatinine ≥2.0 mg/dL, and lactate dehydrogenase ≥300 IU/L were employed to ROC analyses, that revealed an area under the curve of 0.819 for these significant prognostic factors.
(TIF)

S1 Table Pearson correlation coefficients between four biomarkers. (DOCX)

S2 Table Pearson correlation coefficient between blood urea nitrogen and serum creatinine. (DOCX)
