# Correction: Host Factors and Biomarkers Associated with Poor Outcomes in Adults with Invasive Pneumococcal Disease

**DOI:** 10.1371/journal.pone.0153109

**Published:** 2016-03-31

**Authors:** Shigeo Hanada, Satoshi Iwata, Kazuma Kishi, Miyuki Morozumi, Naoko Chiba, Takeaki Wajima, Misako Takata, Kimiko Ubukata, The Invasive Pneumococcal Diseases Surveillance Study Group

There is an error in the Table 3. Within the column labeled “OR (95% CI)”, the values are incorrect for the row labeled “Non-vaccine serotypes”. The correct values are: 1.5 (0.8–3.0). Please see the corrected Table 3 here.

**Table 3:** Risk factors associated with mortality within 28 days among patients with invasive pneumococcal disease by multivariate logistic regression analysis.

Characteristic	Survived (n = 384)	Died (n = 122)	Multivariate analysis ^e^	*p* value ^f^
	n (%)	n (%)	OR (95% CI)	
**Age groups**				
<50 y	45 (11.7)	5 (4.1)	1	-
50–64 y	87 (22.7)	18 (14.8)	1.6 (0.5–5.6)	NS
65–79 y	155 (40.4)	51 (41.8)	3.0 (0.9–9.6)	NS
≥80 y	97 (25.3)	48 (39.3)	6.5 (2.0–21.6)	.002
**Underlying disease**				
Liver disease^a^	33 (8.6)	17 (13.9)	3.5 (1.6–7.8)	.002
Lung disease^b^	34 (8.9)	4 (3.3)	0.4 (0.1–1.4)	NS
**Mechanical ventilation**	52 (13.5)	40 (32.8)	3.0 (1.7–5.6)	< .001
**Laboratory findings** ^c^				
WBC (<4.0×10^3^ cells/μL)	39 (10.2)	41 (33.6)	6.9 (3.7–12.8)	< .001
Cr (≥2.0 mg/dL)	46 (12.1)	44 (36.4)	4.5 (2.5–8.1)	< .001
LDH (≥300 IU/L)	118 (32.1)	61 (53.0)	2.4 (1.4–4.0)	.001
**Serotype** ^d^				
PCV7 serotypes	139 (36.2)	44 (36.1)	1	-
PCV13 and PPSV23 serotypes	182 (47.4)	50 (41.0)	0.8 (0.5–1.5)	NS
Non-vaccine serotypes	60 (15.6)	20 (23.0)	1.5 (0.8–3.0)	NS

^a^ Includes chronic hepatitis or cirrhosis that was secondary to alcohol abuse or viral infection.

^b^ Includes chronic obstructive pulmonary disease, bronchial asthma, interstitial lung disease, and bronchiectasis.

^c^ Laboratory findings; WBC, white blood cell count; Cr, creatinine; LDH, lactate dehydrogenase.

^d^ Serotype; PCV7, 7-valent pneumococcal conjugate vaccine; PCV13, 13-valent pneumococcal conjugate vaccine; PPSV23, 23-valent pneumococcal polysaccharide vaccine.

^e^ Multivariate analysis; OR, odds ratio; CI, confidence interval.

^f^ NS, not significant (*p* ≥ .05).
