# Supplementary material for: Host Factors and Biomarkers Associated with Poor Outcomes in Adults with Invasive Pneumococcal Disease
Source: PLoS One. 2016 Jan 27;11(1):e0147877. doi: 10.1371/journal.pone.0147877 (PMC4731463; doi:10.1371/journal.pone.0147877)
Supplement: S1 Fig — Among 1317 patients with IPD who were admitted to 341 hospitals throughout Japan between April 2010 and March 2013, adults at least 18 years old numbered 715. Patients with meningitis (n = 127) and focal infections such as arthritis, cellulitis, and spondylitis, that were beyond the limits of this survey of IPD (n = 30) were not considered and those with insufficient clinical data (n = 52), were also excluded from this study. After such exclusions, 506 remaining patients followed for 28 days after admission. (DOCX) [file pone.0147877.s001.docx]

1,317 patients were enrolled

602 were <18 years of age

715 patients underwent screening

209 were excluded

127 had meningitis

30 had other disease

52 had insufficient data

506 patients were included in the 28-day analysis
