# Supplementary figures and images for: Host Factors and Biomarkers Associated with Poor Outcomes in Adults with Invasive Pneumococcal Disease
Source: PLoS One. 2016 Jan 27;11(1):e0147877. doi: 10.1371/journal.pone.0147877 (PMC4731463; doi:10.1371/journal.pone.0147877)

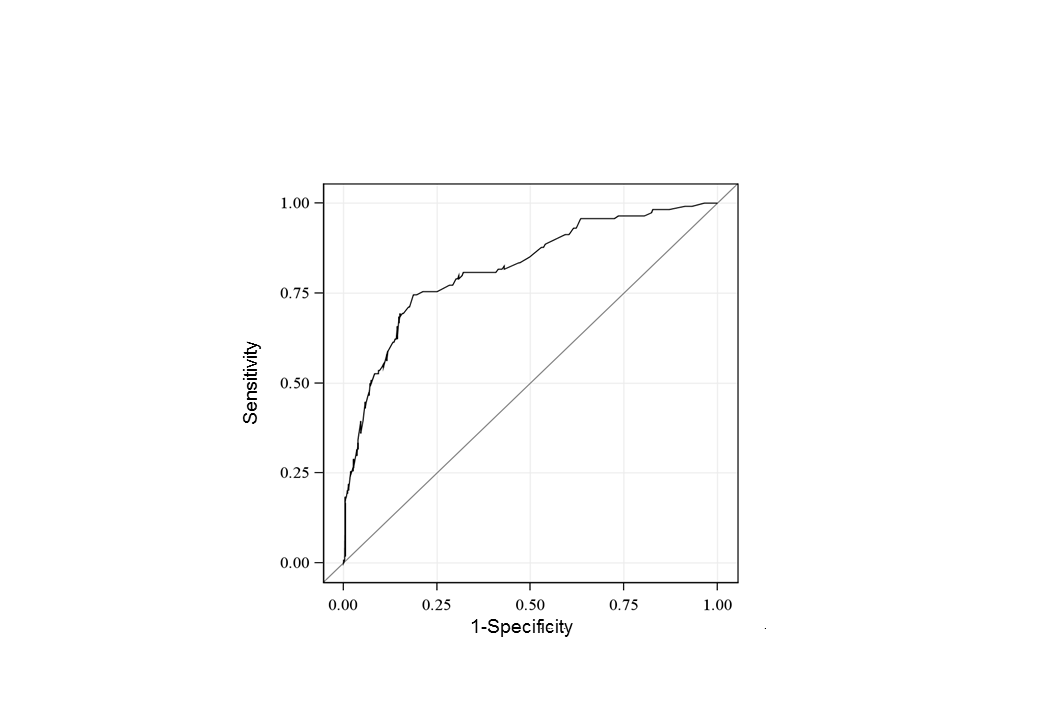

Supplement: S2 Fig — Predictors of age ≥80 years, underlying liver disease, mechanical ventilation, white blood cell count <4000 cells/μL, creatinine ≥2.0 mg/dL, and lactate dehydrogenase ≥300 IU/L were employed to ROC analyses, that revealed an area under the curve of 0.819 for these significant prognostic factors. (TIF) [file pone.0147877.s002.tif]
