# Supplementary material for: Host Factors and Biomarkers Associated with Poor Outcomes in Adults with Invasive Pneumococcal Disease
Source: PLoS One. 2016 Jan 27;11(1):e0147877. doi: 10.1371/journal.pone.0147877 (PMC4731463; doi:10.1371/journal.pone.0147877)
Supplement: S1 Table — (DOCX) [file pone.0147877.s003.docx]

| Biomarker ^a^ | AST | ALT | CK | LDH |
| --- | --- | --- | --- | --- |
| AST |  |  |  |  |
| CC | 1 | 0.956 | 0.654 | 0.978 |
| *p* value | - | <0.001 | <0.001 | < 0.001 |
| ALT |  |  |  |  |
| CC | 0.956 | 1 | 0.657 | 0.930 |
| *p* value | <0.001 | - | <0.001 | <0.001 |
| CK |  |  |  |  |
| CC | 0.654 | 0.657 | 1 | 0.605 |
| *p* value | <0.001 | <0.001 | - | <0.001 |
| LDH |  |  |  |  |
| CC | 0.978 | 0.930 | 0.605 | 1 |
| *p* value | <0.001 | <0.001 | <0.001 | - |

^a^ Abbreviations; AST, aspartate aminotransferase; ALT, alanine aminotransferase;

CK, creatine kinase; LDH, lactate dehydrogenase; CC, correlation coefficient
