# Supplementary material for: Host Factors and Biomarkers Associated with Poor Outcomes in Adults with Invasive Pneumococcal Disease
Source: PLoS One. 2016 Jan 27;11(1):e0147877. doi: 10.1371/journal.pone.0147877 (PMC4731463; doi:10.1371/journal.pone.0147877)
Supplement: S2 Table — (DOCX) [file pone.0147877.s004.docx]

| Biomarker ^a^ | BUN | Cr |
| --- | --- | --- |
| BUN |  |  |
| CC | 1 | 0.647 |
| *p* value | - | <0.001 |
| Cr |  |  |
| CC | 0.647 | 1 |
| *p* value | <0.001 | - |

^a^ Biomarker; BUN, blood urea nitrogen; Cr, creatinine; CC, correlation coefficient
